# Retina‐Inspired Bi‐Based Terahertz Photonic Neuromorphic Devices

**DOI:** 10.1002/advs.75145

**Published:** 2026-04-02

**Authors:** Pujing Zhang, Donggang Xie, Longyu Shi, Mengyuan Wang, Huiwen Shi, Yu Wu, Menglei Li, Guangwei She, Peijie Wang, Wensheng Shi, Cunlin Zhang, Kuijuan Jin, Guozhen Yang, Qingli Zhou, Chen Ge

**Affiliations:** ^1^ Key Laboratory of Terahertz Optoelectronics Ministry of Education and Beijing Advanced Innovation Center for Imaging Theory and Technology Department of Physics Capital Normal University Beijing P. R. China; ^2^ Beijing National Laboratory for Condensed Matter Physics Institute of Physics Chinese Academy of Sciences Beijing P. R. China; ^3^ Key Laboratory of Photochemical Conversion and Optoelectronic Materials Technical Institute of Physics and Chemistry Chinese Academy of Sciences Beijing P. R. China

**Keywords:** 2D material, neuromorphic device, terahertz, van der waals heterojunction

## Abstract

The human retina capable of extracting key feature information is a crucial sensory element in the visual system. Constructing bionic devices with multitype feedback to emulate the retina behaviors in complex environments has been a persistent pursuit to broaden the visual range. However, the definition and regulation of synaptic weights persist as a bottleneck problem in the terahertz (THz) devices to achieve neuromorphic function. Here, we have proposed bismuth‐based THz photonic neuromorphic devices with picosecond short‐term plasticity and constructed the THz‐optical neural network (THz‐ONN) to imitate retina function. Crucially, the weight embodied by THz photoresponse can be precisely regulated via incremental optical pulses, delivering an incredibly simple yet powerful approach that heralds systems with a continuously variable plasticity. Further development of diverse neuromorphic devices for various scenarios could be realized through the combination of band alignment engineering and substrate effects to control photocarrier transport. The corresponding neuromorphic computing based on THz‐ONN indicates the high recognition accuracy of hardware. The present study provides an exciting paradigm for the realization of THz neuromorphic devices and opens an avenue for mimicking biological sensory system.

## Graphical abstract

Combined with effective band alignment and substrate engineering, bismuth materials are introduced to form terahertz photonic bio‐inspired devices with picosecond short‐term plasticity to enable multi‐scene visual perception. Thus obtained hardware through the terahertz optical neural network (THz‐ONN) demonstrates high recognition accuracy, providing an exciting paradigm for the development of novel neuromorphic devices.

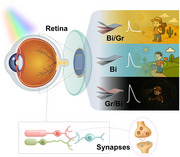

## 1 Introduction

The human visual system plays a crucial role in our interactions with the environment, accounting for more than 80% of our perceived external information [1, 2, 3, 4]. The retina can convert light stimuli into nerve impulses and transmit them to our brain through synapses for further processing [5]. The transmission of the above information is regulated by synapses between neurons, and the communication strength at these junctions determines the synaptic weights [6, 7]. Inspired by the retina, building devices with biomimetic functionality holds great prospects in simulating brain‐like neuromorphic computation and is regarded as a key research line for future artificial intelligence [8, 9]. Wherein, photonic synapses integrate the dual functions of optical signal detection and information processing with ultrahigh propagation speed, high bandwidth, and low crosstalk [6, 10, 11, 12]. More importantly, synaptic devices modulated by photonic signals are in favor of imitating retinal neurons in real eyes, resulting in bridging the gap between brain computing and visual systems [11, 13, 14]. Recently, bio‐inspired vision sensors with synaptic characteristics have been developed in ultraviolet, visible, and infrared spectral ranges, capable of color recognition, image preprocessing, and nociceptive behavior simulation [2, 3, 4, 9, 11, 12, 13, 14, 15, 16]. It is widely recognized that the retinal mechanism is to encode stimulus into synaptic weight to achieve functional diversity. Developing retina‐inspired terahertz (THz) neuromorphic devices is highly important for transcending the biological spectral limit. However, such research is still lacking in the THz band due to unresolved fundamental issues, such as definition of the necessary parameters for neuromorphic computing and establishment of effective modulation methods.

Among various optical wavebands used for neuromorphic devices, the THz radiation offers several special advantages. First, it uniquely bridges the gap between microwave and infrared regimes. This special waveband has a wide spectral range and good directionality, which is suitable for high‐speed data transmission and provides an ideal platform to construct all‐optical neuromorphic systems [17, 18, 19, 20]. Moreover, THz waves have garnered significant attention in biophysics due to their biological safety with low photon energy at the meV level [21, 22, 23]. This ensures they do not damage the molecular structure of biological samples, making them non‐ionizing and thus highly suitable for bio‐applications [21, 24]. In particular, transient THz spectroscopy could capture the many‐body interactions of materials and monitor real‐time dynamics with sub‐picosecond temporal resolution under photoexcitation, providing robust support to develop THz photonic devices [25, 26]. It is known that current photonic or optoelectronic neuromorphic devices typically operate on the second or millisecond timescale. In contrast, this traditional timescale does not reflect the complexity of biological systems since many critical responses of photoreceptor cells are ultrafast and transient, suggesting that the demands of these fast biological reactions cannot be satisfied by the existing devices [27, 28, 29, 30]. Therefore, the construction of THz bio‐inspired devices can extend neuromorphic computing into this spectral regime and provide a novel paradigm for ultra‐vision sensors. Despite these prospects, the advancement of those photonic devices is constrained by the undefined synaptic weights and unclear regulation pathways. For the realization of a practicable THz neuromorphic device, not only the selection of functional materials with multistate photoresponse to define computational weight, but also a device whose weight is precisely controlled by the suitable pulse is crucial. Recently, 2D materials have attracted extensive interest since they exhibit novel and intriguing properties with potential applications [31, 32]. In particular, bismuth (Bi) nanomaterials are the ideal candidates for fabricating THz visual hardware due to the excellent air stability, high carrier mobility, strong light‐matter interaction, and tunable photoresponse [33, 34, 35]. Additionally, van der Waals heterojunction is more flexible in fabrication and integration than traditional heterojunction, thus offering a platform for utilizing substrate effects to effectively regulate material characteristics through the carrier transport at the heterointerface [36, 37, 38]. These studies inspire us to exploit novel Bi‐based THz neuromorphic devices.

Here, we propose the retina‐inspired THz photonic devices capable of picosecond short‐term plasticity (PSTP) under the control of light pulses to realize multi‐scene visual perception. Specifically, three intelligent devices are fabricated based on Bi materials through the synergy between band alignment engineering and substrate effects to regulate photogenerated carrier dynamics, thereby inducing significantly discriminative THz photoresponses. Based on those distinct properties, we demonstrate three retinomorphic devices that can encode optical stimuli into modulation signals, showing the capability of simulating retinal function in different scenarios of deserts, lawns, and mines. Our constructed retina‐inspired neuromorphic devices are further incorporated as the input neurons of a 3‐layer THz‐optical neural network (THz‐ONN), achieving around 96% accuracy on MNIST‐based stimulus image classification. Our work provides a flexible strategy to develop novel biomimetic visual systems, laying a solid foundation for the design of THz neuromorphic devices.

## 2 Results and Discussion

### 2.1 Retina‐Inspired THz Neuromorphic Devices Based on Bi Materials

**FIGURE 1:**
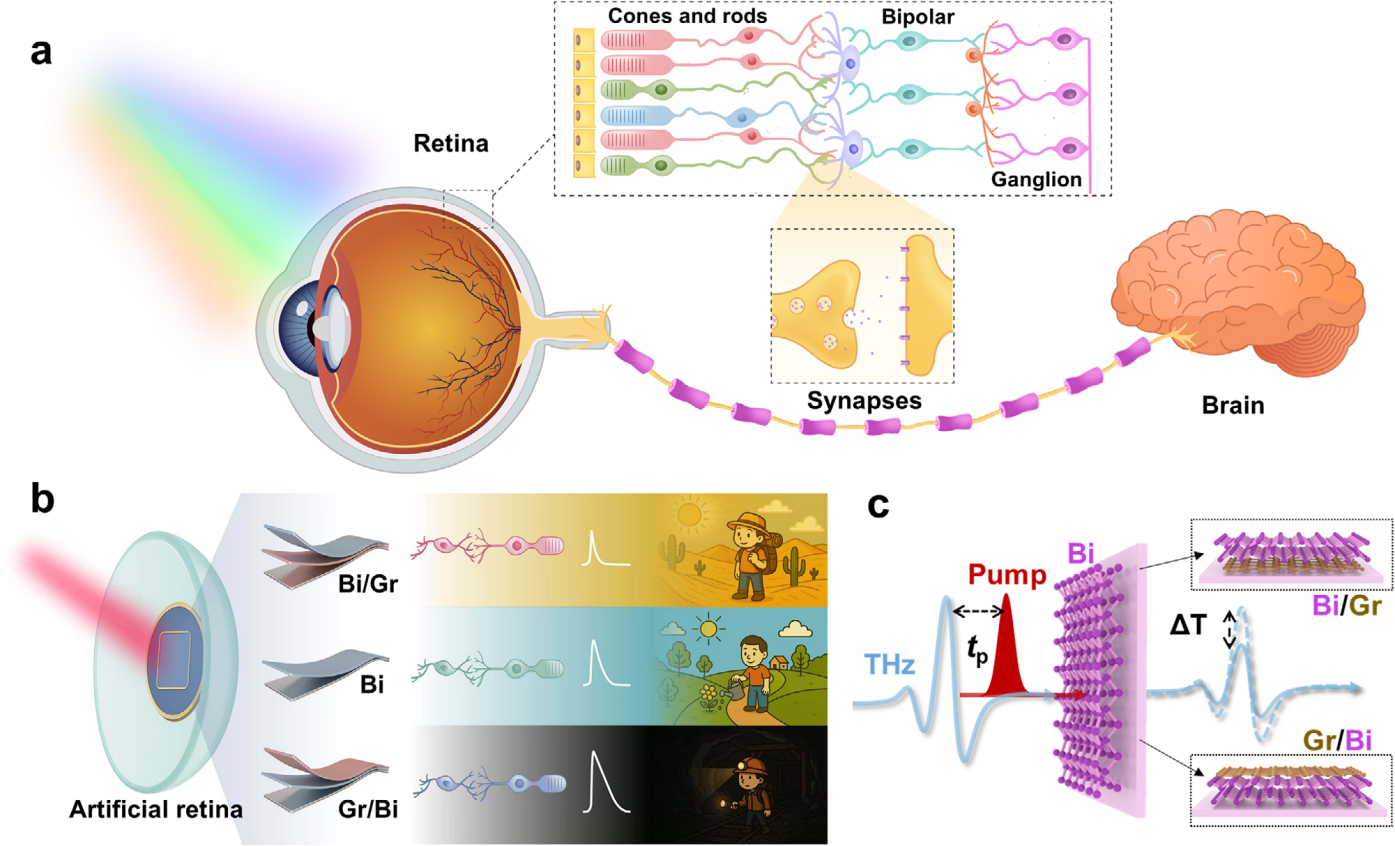
Comparison of human visual system and Bi‐based THz neuromorphic devices. (a) Schematic workflow of the human visual system. (b) Illustration of Bi‐based artificial retinas applied in diverse work scenarios based on the ERG signals of the human retina. (c) THz measurement scheme of Bi nanofilm and two heterostructures (Bi/Gr and Gr/Bi).

Our work is inspired by the biological retina, where distinct types of photoreceptor cells are specialized to process different illumination scenarios [1, 2, 3, 4, 16, 39, 40, 41]. Figure 1a shows the perception and processing of external visual information in the human visual system. The photoreceptor cells in the retina are in charge of perceiving light stimuli and converting them into electrical signals, which travel through the optic nerves to reach the visual cortex. Specifically, the input visual signals are sensed and preprocessed by photoreceptors (rods and cones) and horizontal cells in the retina. The rod cells are responsible for scotopic (dim light or night) vision due to their superior photosensitivity, whereas cone cells provide photopic (bright light or daylight) vision characterized by higher spatial, temporal, and spectral resolution [42, 43, 44]. Then, the bipolar cells receive signals from the photoreceptors and transmit them to the ganglion cells [40]. The transmission of the above information is regulated by horizontal and bipolar cells through synapses between neurons. Therefore, it is of great significance to develop the bio‐inspired visual system in hardware with synaptic features that can adapt to complex environments. It is known that retinal electroretinogram (ERG), which can reflect the response of the retina cells to light stimuli, exhibits variable parametric characteristics under different conditions [45, 46, 47]. Here, we demonstrate a correspondence between ERG characteristics and material photoresponse properties characterized by THz amplitude modulation and carrier relaxation time. In our constructed visual systems, three Bi‐based neuromorphic devices, whose photoresponses can correspond to the different profiles of ERG signal, are proposed to emulate the retina behaviors in different work scenarios, namely deserts, lawns, and mines (Figure 1b) [45, 46, 47]. Wherein, the desert scenario represents a light environment requiring photopic vision with reduced ERG amplitude and narrow time domain to prevent overstimulation, similar to the photoresponse with small magnitude and fast relaxation in Bi/graphene (Bi/Gr) device. Conversely, the mine scenario represents a dim environment requiring scotopic vision to obtain high‐amplitude ERG signal to maximize sensitivity, matching our Gr/Bi device with high magnitude and slow relaxation. The lawn represents an intermediate state, corresponding to the optical property of Bi nanofilm. Our experimental scheme is illustrated in Figure 1c using optical pump‐THz probe system to investigate the optical responses of Bi‐based THz devices [26]. The right panel presents the side views of the Bi/Gr (upper) and Gr/Bi (lower) heterostructures fabricated with Bi and monolayer Gr on the fused silica (FS) substrates. We monitored the peak value of the THz waves with variable time delays between optical pump and THz probe, which allowed photocarrier dynamics to be probed (see details in Experimental Methods).

### 2.2 Transient Dynamics Properties of Three Bi‐Based Neuromorphic Devices

**FIGURE 2:**
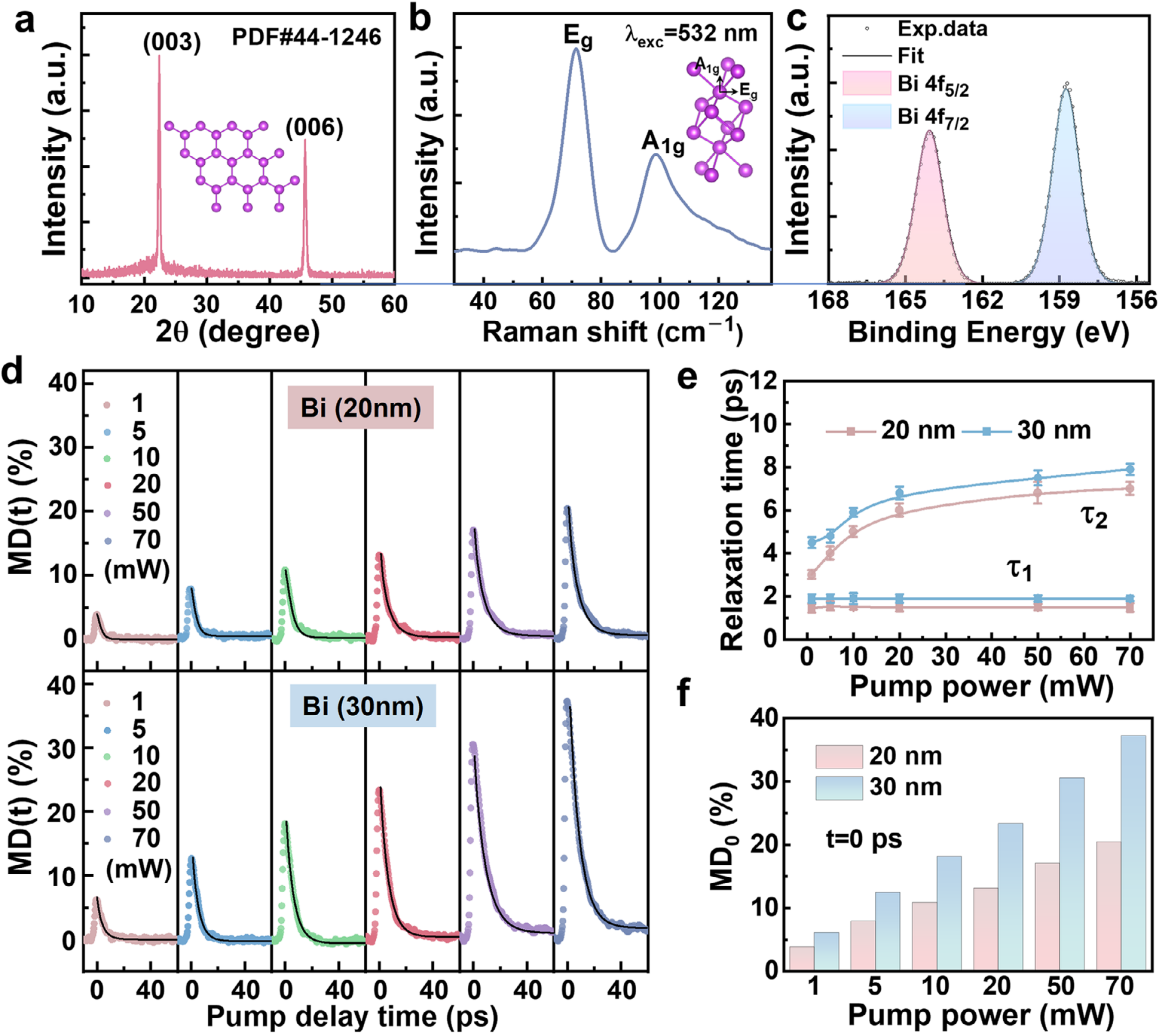
Transient dynamics properties of Bi. (a) XRD pattern, (b) Raman and (c) XPS spectra Bi nanofilm. (d) *MD*(*t*) for Bi (20 nm) and Bi (30 nm) under various pump powers. Black solid lines represent the biexponential fitting curves. (e) Pump power dependence of the extracted relaxation times (*τ*_1_ and *τ*_2_). f) *MD*_0_ corresponding to *MD*(*t*) at pump delay time of 0 ps for Bi nanofilms.

Large‐area Bi nanofilms were deposited by the electron beam evaporation method on FS substrates (see details in Experimental Methods). The *x*‐ray diffraction (XRD) pattern of Bi (30 nm) is given in Figure 2a, showing that the characteristic peaks are highly coincident with the standard spectrum (PDF 44–1246). Particularly, strong diffraction peaks (003) and (006) indicate the preferred orientation along the (001) family of planes in a hexagonal structure. Bi nanofilm crystallizes with a rhombohedral layered *β*‐phase structure, which is the stable allotropic form under atmospheric pressure. The inset of Figure 2a displays a top view of hexagonal Bi with a honeycomb structure. A typical Raman spectrum of Bi shown in Figure 2b has two first‐order Raman bands at 71.4 and 98.5 cm^−1^ associated with two characteristic optical phonon modes *E_g_* (in‐plane) and *A*_1_ *_g_* (out‐of‐plane) of the rhombohedral lattice, respectively. The side view in the inset reveals a buckled multilayer structure. Figure 2c presents the survey *x*‐ray photoelectron spectroscopy (XPS) spectrum of Bi with the fitted 4f regions. Two obvious peaks with binding energies of 159.1 and 164.4 eV can be observed, which are assigned to Bi 4f_7/2_ and Bi 4f_5/2_, respectively [33].

The measured THz transient dynamics are demonstrated in Figure 2d for Bi (20 nm) and Bi (30 nm). The relative change is quantified by the modulation depth *MD*, defined as a function of pump delay time *t*: *MD*(*t*) = −Δ*T*/*T*_0_ = −(*T*−*T*_0_) /*T*_0_, where *T*_0_ and *T* are the THz peak values before and after the photoexcitation. It is found that *MD*(*t*) first exhibits rapid change owing to the photogenerated carriers, and then recovers to the initial state. We fitted decay processes using biexponential functions and obtained relaxation times (fast component *τ*_1_ and slow component *τ*_2_), as demonstrated in Figure 2e. The decay process can be divided into two distinct components. Wherein, the fast relaxation component *τ*_1_ is due to the energy relaxation of the carriers, while the slow component *τ*_2_ depicts the lifetime of the carriers. For Bi (20 nm), *τ*_1_ is about 1.5 ps and independent of the pump power, while *τ*_2_ slows down from 3.0 to 6.9 ps with the increased pump power. For Bi (30 nm), *τ*_1_ is about 1.6 ps and *τ*_2_ varies from 4.5 to 7.8 ps. This pump‐independent trend of *τ*_1_ reveals that the fast relaxation process is mainly assisted by electron‐phonon scattering [48, 49]. On the other hand, variable *τ*_2_ could be attributed to trap‐assisted recombination, as it increases with pump power. Moreover, we have extracted the corresponding *MD*(*t*) at pump delay time of 0 ps as *MD*_0_ from Figure 2d. As shown in Figure 2f, *MD*_0_ values for Bi (20 nm) and Bi (30 nm) all increase with the pump power. Furthermore, it can be seen that those nanofilm modulators are excitable even at the pump power of 1 mW, with *MD*_0_ values of 3.8% and 6.1%, respectively. At 70 mW, *MD*_0_ values increase to 20.3% and 37.9%, respectively. These results imply that the relaxation times of Bi (20 nm) and Bi (30 nm) are close but the difference in *MD*_0_ is quite remarkable. For comparison, we have also provided transient dynamics properties of Bi (50 nm) (Figure S1). It can be seen that *MD*_0_ of Bi (50 nm) is almost the same as that of Bi (30 nm) but with a low transmission. Thus, Bi (30 nm) is the optimal candidate to improve modulation characteristics in our subsequent studies, providing sufficient *MD*_0_ with a larger dynamic range for effectively discriminative encoding.

**FIGURE 3:**
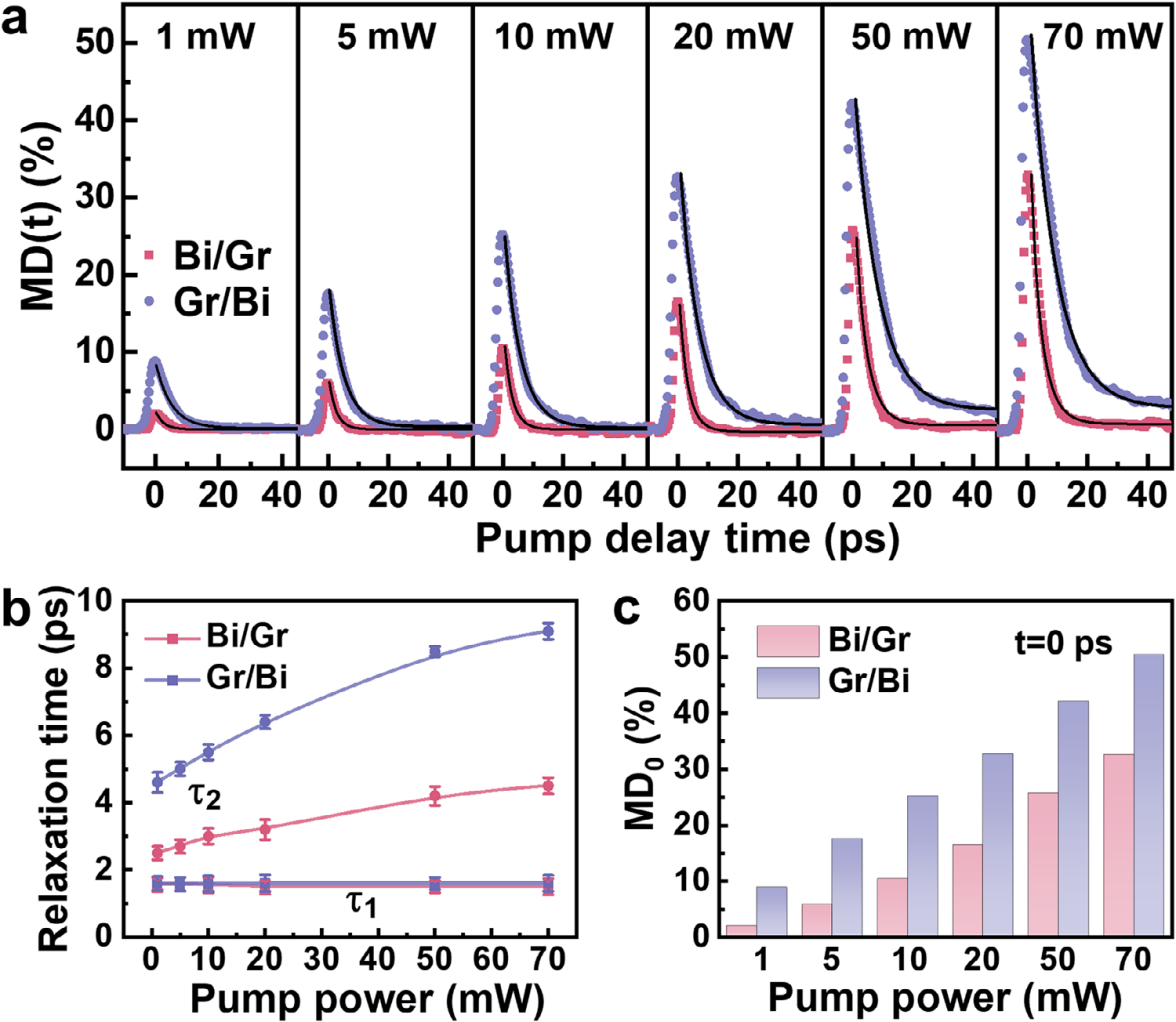
Transient dynamics properties of Bi‐based heterojunctions. (a) *MD*(*t*) under various pump powers, (b) pump power dependence of the extracted relaxation times and (c) *MD*_0_ for Bi/Gr and Gr/Bi heterojunctions, respectively.

Since the heterointerface could tune the photoelectric conversion efficiency, the Bi‐based heterojunction is expected to further improve the device performance. Here, we have studied the THz responses of the heterojunctions formed by Bi (30 nm) and Gr (monolayer) in different stacking orders. The Raman spectrum of latter with G‐ and 2D‐bands confirms the monolayer nature of Gr (Figure S2) [36]. The transient THz dynamics of Gr exhibits the negative THz photoconductivity, which is ascribed to the enhancement of carrier scattering rate overtaking the increase in Drude weight (Figure S3 and Table S1). Figure 3a demonstrates *MD*(*t*) for Bi/Gr and Gr/Bi heterojunctions under 800 nm pump with various powers. These heterojunctions present positive THz photoconductivity behaviors and their relaxation processes still have two distinct decay components. It is obvious that the stacking order significantly influences the transient dynamics. Further extraction of the relaxation times of Bi/Gr and Gr/Bi is presented in Figure 3b. Their fast components *τ*_1_ are nearly the same with the value of about 1.7 ps and independent of pump power. Meanwhile, *τ*_2_ varies from 2.5 to 4.5 ps for Bi/Gr and 4.6 to 9.1 ps for Gr/Bi with the increased pump power from 1 to 70 mW. The trend of *τ*_1_ and *τ*_2_ in these heterostructures is consistent with those of Bi nanofilm. Intriguingly, compared to that of Bi (*τ*_2_ = 7.8 ps@70 mW), the stacking order of Bi/Gr can improve the modulation speed by nearly two times. On the other hand, the extracted *MD*_0_ of Bi/Gr and Gr/Bi all increase with pump power, as shown in Figure 3c. It is found that the *MD*_0_ values of Bi/Gr and Gr/Bi are 2.0% and 8.8% at 1 mW, followed by 32.6% and 50.4% at 70 mW, respectively. The *MD*_0_ values of two heterojunctions exhibit huge difference. Compared to that of Bi (*MD*_0_ = 37.9%@70 mW), the stacking order of Gr/Bi can enhance *MD*_0_ by 30%. Our results indicate that the stacking order of the materials on demand enables the selective optimization of the modulation speed or depth. It is known that compared with ERG signals under dark adaptation, light‐adapted signals are characterized by reduced amplitude and narrow time domain [45, 46, 47]. As shown in Figure 1b, the transient THz dynamics of Bi/Gr heterojunction with the relatively small *MD*_0_ value and fast speed can reflect ERG signals of human electroretinogram in bright environment. Therefore, based on the above device characteristics and inspired by the working principle of the human retina, Bi/Gr heterojunction, Bi nanofilm, and Gr/Bi heterojunction can serve as the artificial retinas for working in deserts, lawns, and mines, respectively.

### 2.3 Working Principle of the Devices

**FIGURE 4:**
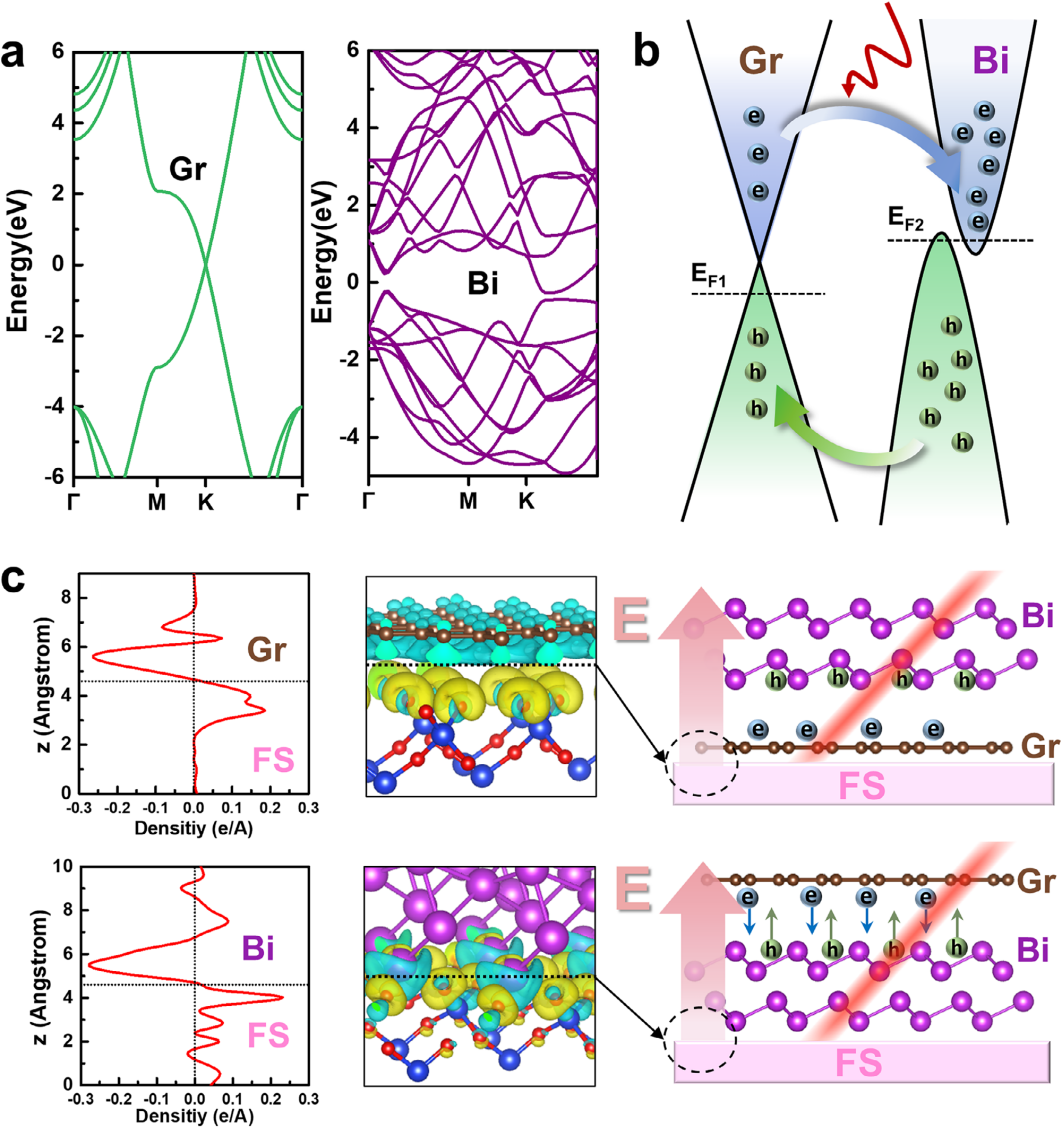
Substrate‐induced carrier dynamics in Bi/Gr and Gr/Bi heterojunctions. (a) Calculated band structures and (b) Energy band diagram of Gr and Bi. (c) Left: Calculated space distribution of the differential charge density for different materials on substrate. Blue (yellow) denotes electron depletion (accumulation). Right: Schematic substrate‐induced electric fields and photocarrier transfer in heterojunctions of Bi/Gr (upper) and Gr/Bi (lower), respectively.

To explore the working principle of devices, we have performed the first‐principles calculations and theoretical analysis. Here, we first give the Dirac‐cone band structure of Gr with density functional theory (DFT) calculations, as given in Figure 4a (see details in the Supporting Information) [50, 51]. Moreover, our calculated valence and conduction bands of Bi overlap with the value of 0.16 eV. Figure 4b illustrates the energy band diagram of Gr and Bi. Wherein, the energy level of the Dirac point of Gr with respect to the vacuum level is −4.5 eV [50]. Our measured Raman results of Gr transferred onto FS indicate the *p*‐doping nature due to blue‐shifting of the G‐band and 2D‐band (Figure S2) [36, 52]. In addition, the reported work function of Bi is 4.32 eV, which is smaller than that of Gr, establishing a built‐in electric field pointing from Bi to Gr [33, 34, 35, 53]. This will result in a potential barrier in Bi‐Gr heterojunctions, which can be verified by surface potential mapping between Gr and Bi (Figure S4). Under photoexcitation, photogenerated electrons in Gr will cross the barrier into Bi through built‐in electric field, while the photogenerated holes are transferred from Bi to Gr. It is assumed that the effective field of the substrate could modify charge transfer process at the heterointerface through stacking order, thereby regulating the optical transient behavior [26, 36, 37]. Hence, we have calculated the plane‐averaged differential charge densities and spatial charge distributions using DFT for Gr on FS and Bi on FS, respectively, as shown in the left panel of Figure 4c. The electron depletion layers at the side of 2D materials reveal that the direction of effective electric field points out of the substrate. After photoexcitation, the electric field introduced by the substrate can suppress the charge transfer process for Bi/Gr, facilitating the recombination of electron‐hole pairs to induce a shorter carrier lifetime and smaller *MD*_0_ value. For Gr/Bi, the substrate effect promotes the transfer process of photocarrier. The enhanced separation of electrons and holes significantly reduces their recombination, leading to an increase of the carrier lifetime as well as the carrier density. Therefore, the *MD*_0_ can be largely improved with the prolonged relaxation times. Our results indicate that through band alignment and substrate engineering, the properties of sensor devices can be effectively regulated for THz neuromorphic computation.

### 2.4 Bi‐Based Neuromorphic Visual Systems

**FIGURE 5:**
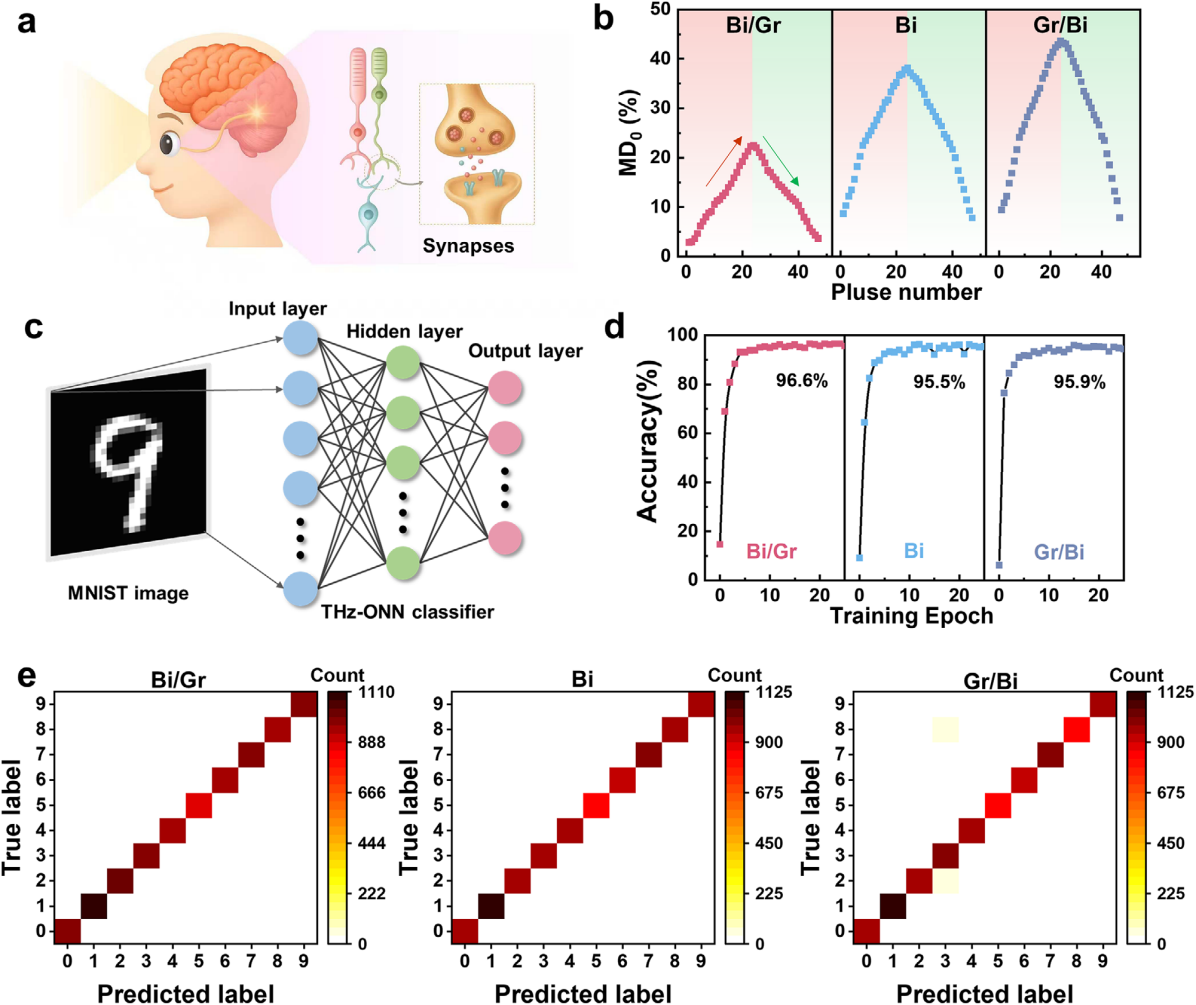
Stimulation recognition based on THz photonic neuromorphic devices. (a) Schematic diagram of the biological visual system to implement stimulus processing. (b) Potentiation and depression synaptic function characteristic behaviors of devices based on Bi/Gr heterostructure, Bi nanofilm, and Gr/Bi heterostructure. (c) Schematic of a THz‐ONN for handwritten digits recognition. (d) Recognition accuracy of devices. (e) Confusion matrix of the classification results of the test dataset.

To examine the device‐to‐device variation, we randomly selected 100 devices from each of the Bi/Gr, Bi, and Gr/Bi samples (Figure S5). The *MD*(*t*) curves of three systems all exhibit the relatively concentrated distribution, reflecting excellent device uniformity. Notably, after removing the optical pulse, the transient evolutions of the *MD*(*t*) for the Bi/Gr, Bi, and Gr/Bi structures indicate the weight does not vanish instantaneously and can be maintained for a duration within tens of picoseconds. Hence, our system exhibits short‐term plasticity at the picosecond scale. This PSTP characteristics of devices is mainly determined by the slow relaxation component since the contribution of the fast component is negligible due to the extremely short duration involved in thermalization and minor proportion. As depicted in Figure 5a, in the human visual system, the retina provides exteroceptive sensations, helping us perceive and preprocess stimuli. Wherein, the photoreceptor cells can extract the information from the outside world and then convert the optical input into an electrical signal for later recognition. To demonstrate the capacity of the devices in neuromorphic computing, the potentiation and depression functions of retina‐inspired neuromorphic devices based on Bi/Gr heterostructures, Bi nanofilms, and Gr/Bi heterostructures were analyzed in Figure 5b. For all‐photonic synapses, potentiation and depression are regulated by modulating parameters of the optical pulses (such as interval, number, or power) to induce distinct physical state changes in the material [6, 10, 11, 12, 54, 55, 56]. Therefore, in our study, we have regulated synaptic weights of three structures by varying the input pump power to achieve the potentiation/depression behavior. Obviously, *MD*_0_ values of the devices change regularly with the applied pulse power. Such symmetric and linear features will improve the performance of neuromorphic computing systems, such as the recognition accuracy in THz‐ONN systems. The above results further validate the practicality and stability of devices at the conventional second on/off pulse intervals (Figure S6). The corresponding *MD*(*t*) curves after the cessation of the 24th incremental pulse still possess the PSTP behaviors (Figure S7). To further demonstrate the potential of our device in retinal preprocessing and THz‐ONN recognition, we simulated and constructed an image classification system for THz pattern recognition based on experimental data. Figure 5c shows the schematic of MNIST‐based stimulus image classification (see details in the Supporting Information). Here, the gray value of each pixel in a handwritten digit image is regarded as a THz stimulus, and 784 sensory neurons are used to sense the THz stimulus and encode it into different *MD*(*t*). The input information encoded as *MD*(*t*) is then read as modulated THz wave and fed into a three‐layer THz‐ONN consisting of 784 input neurons, 300 hidden neurons, and 10 output neurons for processing. The stimuli images can be inferred into 10 different categories after training the network. Figure 5d shows the evolution of the test accuracy during training process of Bi/Gr heterostructure, Bi nanofilm, and Gr/Bi heterostructure, where the classification accuracy after 10 training epochs on the test dataset can reach 96.6%, 95.5%, and 95.9%, respectively. Figure 5e further shows a confusion matrix of the classification results of the 10 000 test images after 10 epochs. The columns here designate the actual labels of stimuli images, while the rows represent the inferred results, and the color bars indicate the number of instances. Most of the test images can be classified correctly, indicating that all of the three types of fabricated artificial retinas can complete complex tasks when applied to different scenarios. We have provided Table S2 to compare reported device properties based on 2D materials. It is found that most existing devices in the ultraviolet, visible, and near‐infrared bands typically operate on second or millisecond timescales. Our work utilizes the unique ultrafast photocarrier dynamics of 2D materials in the THz band to achieve picosecond‐scale transient responses, emulating the visual processing of the retina.

## 3 Conclusions

In summary, we have developed three Bi‐based THz photonic neuromorphic devices with PSTP behaviors as artificial retinas to realize high recognition accuracy, where the synaptic weight is determined by the THz photoresponse and dynamically modulated by incremental pulses. It is found that *MD* of Bi (30 nm) is 37.9% at 70 mW accompanied by the recombination time of 7.8 ps. Intriguingly, the Bi/Gr heterojunction accelerates the modulation speed to 4.5 ps with a reduced *MD*_0_ and the Gr/Bi heterojunction enhances the *MD*_0_ value to 50.4% with extended relaxation time. We have attributed these phenomena to that the separation of photogenerated carriers can be suppressed in Bi/Gr and promoted in Gr/Bi via the substrate effective field, thereby significantly affecting the device properties. Based on above photoresponse characteristics, we applied Bi/Gr heterostructures, Bi nanofilms, and Gr/Bi heterostructures to work in deserts, lawns, and mines, respectively, for further neuromorphic computing. Through THz‐ONN training, the devices exhibit recognition accuracy of 96.6%, 95.5%, and 95.9%, leading to successful pattern classification on stimulus images. This work provides a promising strategy for designing multitype perceptive neuromorphic devices using unique combinations of materials for future intelligent applications.

## 4 Experimental Methods

### 4.1 Sample Preparation of Devices

Bi nanofilms were grown on the FS substrates in an electron‐beam evaporator with an in situ thickness meter. Before the deposition, the substrates were cleaned by ultrasonication in acetone for 10 min and rinsed with isopropyl alcohol and deionized water. The deposition of Bi was initiated from a 99.9% pure Bi source in a boron nitride crucible at a rate of 0.1 nm s^−1^ under 10^−4^ Pa pressure. Annealing was performed in a tube furnace at 373 K under a 100 sccm Ar atmosphere for 0.5 h. Monolayer CVD‐grown Gr from SixCarbon Technology (Shenzhen, China) was transferred onto the prepared samples.

### 4.2 Material Characterization

XRD pattern was performed using a Rigaku SmartLab instrument with a 2*θ* range from 20° to 80° in step of 0.05°. Raman spectrum was analyzed using the alpha300 R microscope under 532 nm laser excitation. XPS measurements were performed on ThermoFisher Scientific ESCALAB 250X under monochromatic Al Kα radiation with an energy of 1486.6 eV.

### 4.3 Optical Pump‐THz Probe Measurements

The 800 nm source beam with 100 fs duration and 1 kHz repetition rate delivered by a Spectra Physics regenerative amplifier can be divided into three paths to generate THz wave, probe the THz signal, and pump the samples, respectively. We first measured the transmitted THz signals by blocking the pump beam. Then, a 1D pump curve can be obtained by scanning the pump delay line with the fixed position of THz generation delay line at the peak of the THz pulse. The spot size of pump beam is about 0.3 cm^2^.

## Conflicts of Interest

The authors declare no conflicts of interest.

## Supporting information




**Supporting File**: advs75145‐sup‐0001‐SuppMat.docx.

## Data Availability

The data that support the findings of this study are available from the corresponding author upon reasonable request.
