# Supplementary material for: Retina‐Inspired Bi‐Based Terahertz Photonic Neuromorphic Devices
Source: Adv Sci (Weinh). 2026 Apr 2:e75145. Online ahead of print. doi: 10.1002/advs.75145 (PMC13325657; doi:10.1002/advs.75145)
Supplement: Supplementary file 1 — Supporting File: advs75145‐sup‐0001‐SuppMat.docx. [file ADVS-9999-e75145-s001.docx]

Supporting Information

Retina-Inspired Bi-Based Terahertz Photonic Neuromorphic Devices

*Pujing Zhang, Donggang Xie, Longyu Shi, Mengyuan Wang, Huiwen Shi, Yu Wu, Menglei Li, Guangwei She, Peijie Wang, Wensheng Shi, Cunlin Zhang, Kui-juan Jin, Guozhen Yang, Qingli Zhou***, Chen Ge**

P. Zhang, L. Shi, M. Wang, H. Shi, Y. Wu, M. Li, Prof. P. Wang, Prof. C. Zhang, Prof. Q. Zhou

Key Laboratory of Terahertz Optoelectronics, Ministry of Education, and Beijing Advanced Innovation Center for Imaging Theory and Technology, Department of Physics, Capital Normal University, Beijing 100048, P. R. China

E-mail: [qlzhou@cnu.edu.cn](mailto:qlzhou@cnu.edu.cn) (Q. Z.)

D. Xie, Prof. K.J. Jin, Prof. G. Yang, Prof. C. Ge

Beijing National Laboratory for Condensed Matter Physics, Institute of Physics, Chinese Academy of Sciences, Beijing 100190, P. R. China

E-mail:  [gechen@iphy.ac.cn](mailto:shegw@mail.ipc.ac.cn)  (C. G.)

Prof. G. She, Prof. W. Shi

Key Laboratory of Photochemical Conversion and Optoelectronic Materials, Technical Institute of Physics and Chemistry, Chinese Academy of Sciences, Beijing 100190, P. R. China

**1.** **Transient THz dynamics and Atomic Force Microscope (AFM) images for Bi films**


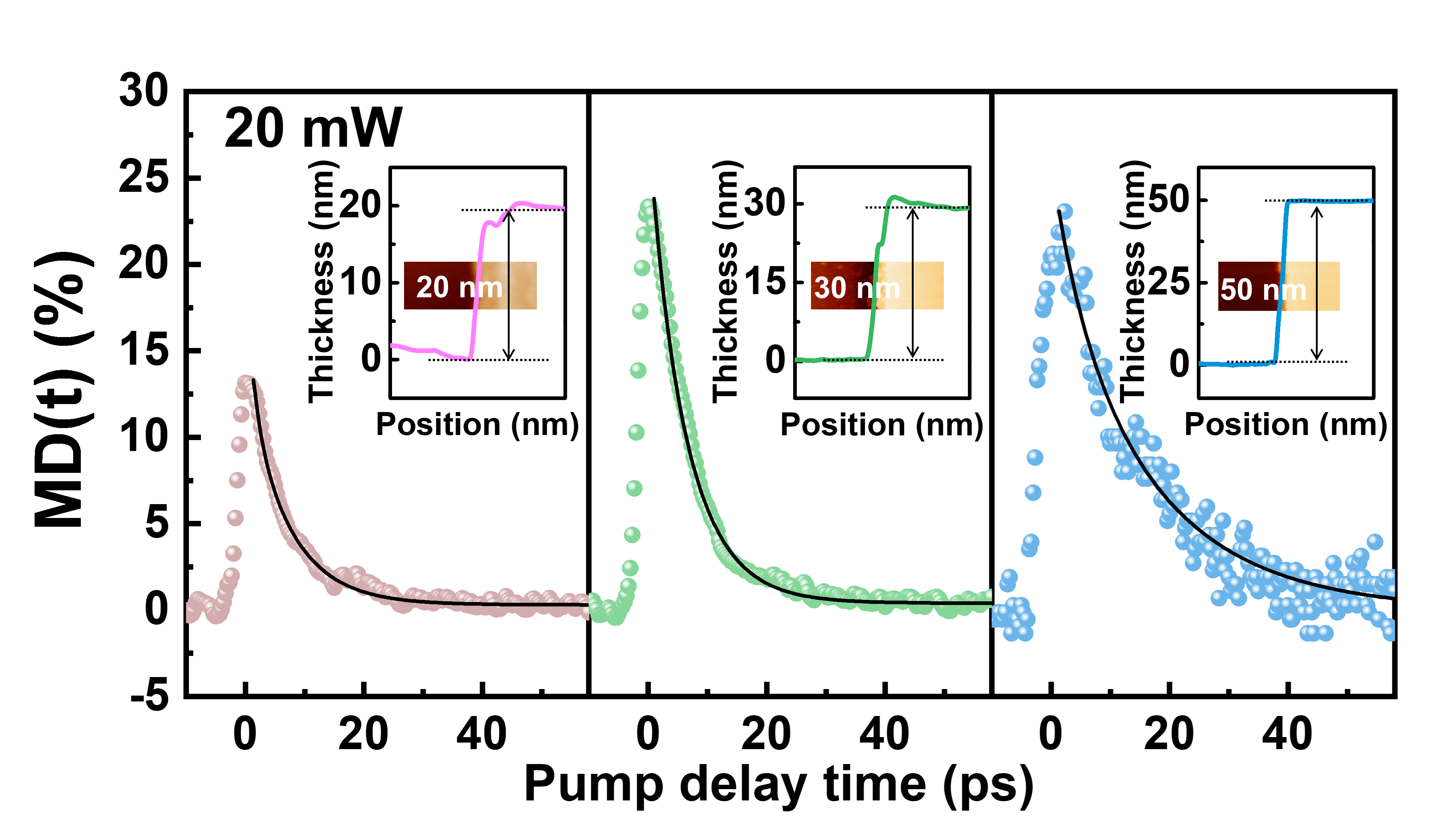


**Figure S1.** Transient THz dynamics for 20, 30, and 50 nm Bi films under 800 nm pump at 20 mW. Inset: AFM images.

As shown in Figure S1, we have presented the transient THz dynamics of Bi nanofilm with different thicknesses and their AFM images, and it can be seen that the thicknesses are around 20 nm, 30 nm, and 50 nm, respectively. The modulation depth of Bi (50nm) is about 23.6%, which is close to that of Bi (30 nm) and much larger than that of Bi (20 nm). Moreover, the time constants for Bi (50nm) are significantly longer for both the fast and slow decays (*τ*_1_=4.2 ps and *τ*_2_=17.5 ps), compared to those of Bi (20 nm) and Bi (30 nm).

**2.** **Raman spectrum and transient THz dynamics for Gr**

**2.1** **Raman spectrum of Gr**

It is known that the two characteristic Raman modes centered at around 1580 and 2650 cm^−1^ in bare Gr are assigned to the G-band and 2D-band.^1,2^ As shown in Figure S2, our measured Raman results excited by a 532 nm laser indicate that the Fermi level of Gr shifts downwards from the Dirac point due to the blue-shifting of G-band and 2D-band.

According to the empirical relation between the G-band positions and the Fermi energy level *E_F_* is *ω_G_* − 1580 cm^−1^ = 42 cm^−1^/eV · |*E_F_*|, we can estimate that the *E_F_* shifts about 94 meV below the Dirac point.^3^ Moreover, the spectrum confirms the high quality of the monolayer graphene. The intensity ratio *I*_2_*_D_*/*I_G_* is approximately 3, and the 2D-band exhibits a sharp, symmetric Lorentzian profile, which are distinct fingerprints of monolayer Gr.^2^


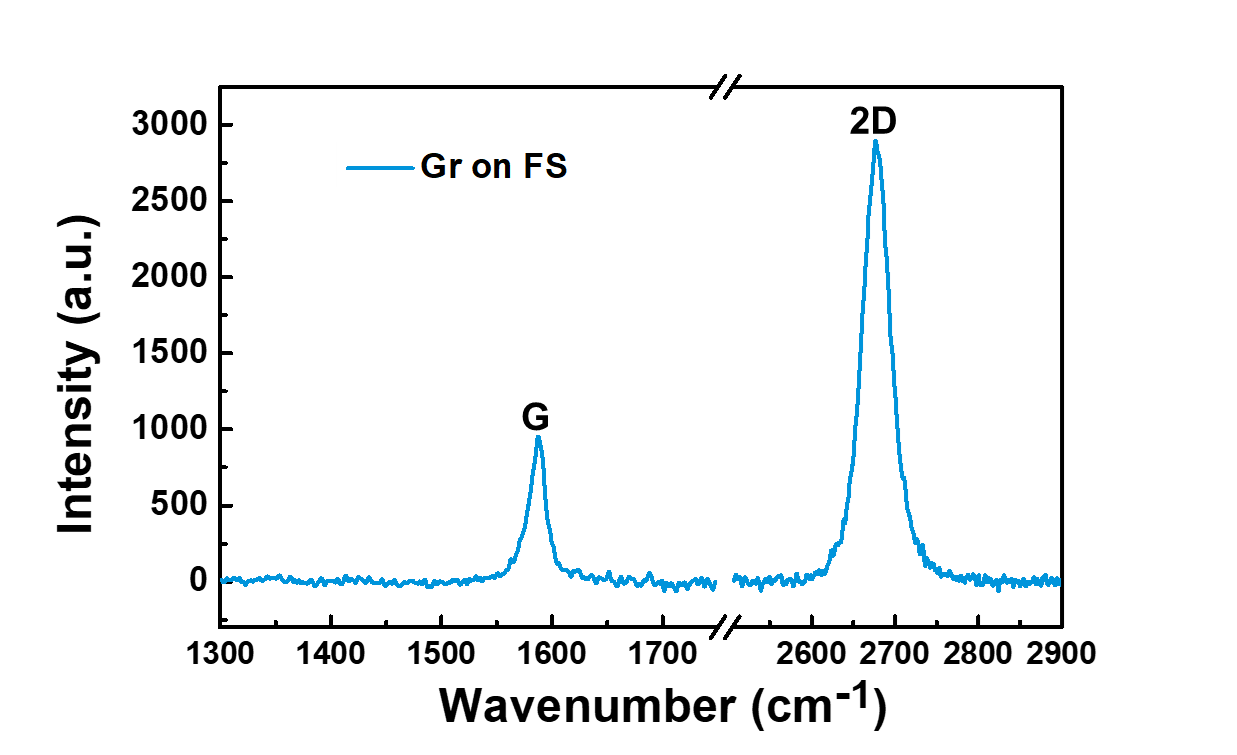


**Figure S2.** Measured Raman spectrum of Gr on FS.

**2.2 Transient THz dynamics of Gr under 800 nm pump**


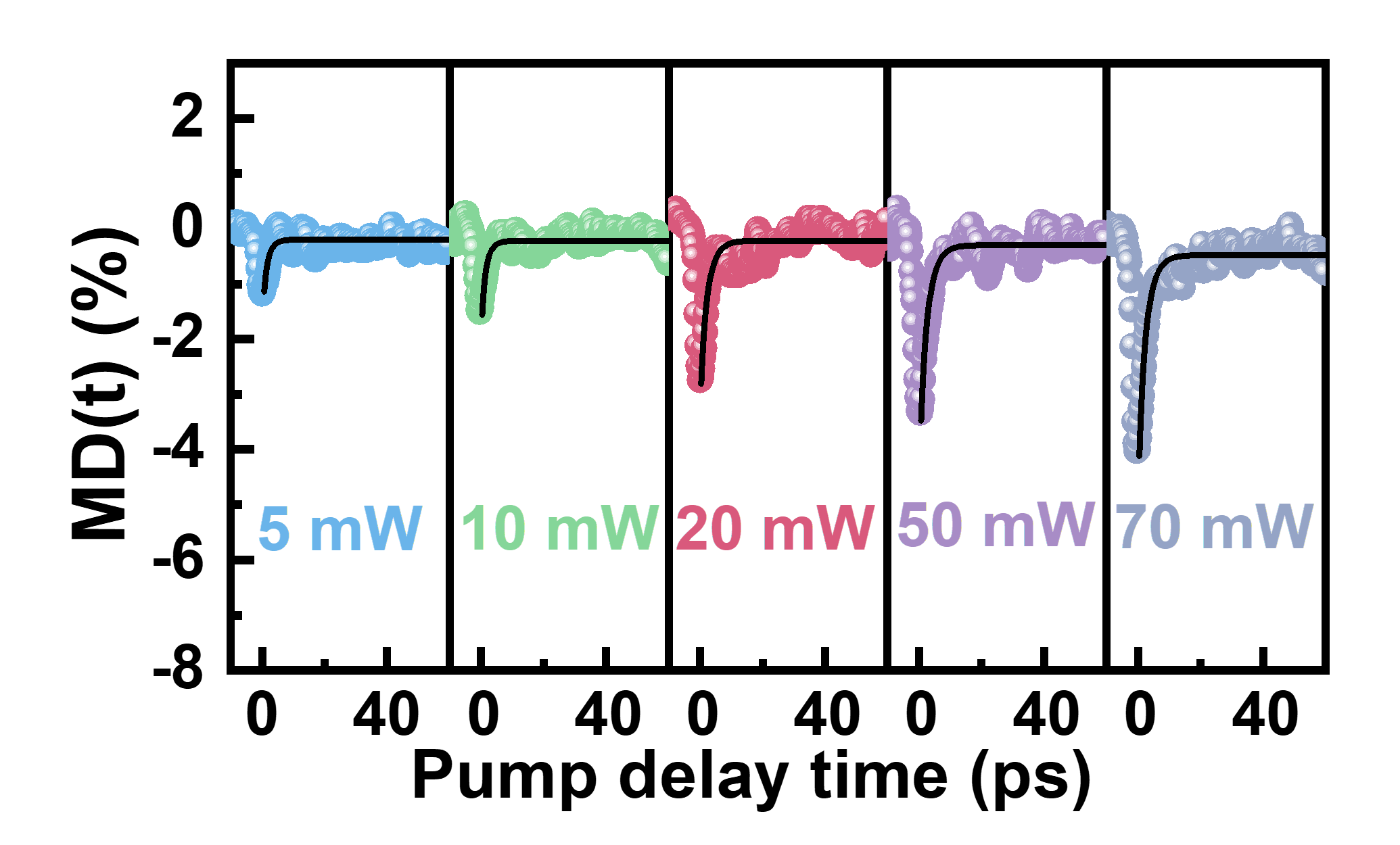


**Figure S3.** Transient THz dynamics of Gr under various pump powers.

**Table S1**. Fitting parameters of transient THz dynamics of Gr

| Power (mW) | A_1_ | A_2_ | *τ*_1_ (ps) | *τ*_2_ (ps) |
| --- | --- | --- | --- | --- |
| 5 | 0.40 | 1.10 | 0.80 | 2.20 |
| 10 | 0.40 | 1.20 | 1.10 | 2.40 |
| 20 | 2.50 | 1.50 | 1.20 | 4.00 |
| 50 | 2.90 | 1.68 | 1.48 | 4.20 |
| 70 | 3.00 | 1.70 | 1.50 | 4.30 |

We measured the *MD*(*t*) of Gr under the 800 nm pump excitation shown in Figure S3, exhibiting the negative THz photoconductivities. We have extracted the relaxation time constants (*τ*_1_ and *τ*_2_) via biexponential fitting and given the parameters in Table S1.

**3.** **First-principles calculations**

The calculations based on DFT were performed by using the projected augmented wave (PAW) method as implemented in Vienna Ab Initio Simulation Package (VASP). A plane-wave cutoff energy of 450 eV was employed. The valence electron configurations of Bi, C, Si, and O were 6*s*6*p*, 2*s*2*p*, 3*s*3*p*, and 2*s*2*p*, respectively. For the calculations of Bi and Gr, the strongly constrained and appropriately normed (SCAN) functional was used to describe the exchange-correlation interactions for the structural optimizations. Based on the relaxed structures, we applied hybrid Heyd-Scuseria-Ernzerhof (HSE) functional to calculate the electronic band structures since the electronic structure would be well described by using the hybrid DFT with spin orbit coupling (SOC). Meanwhile, a Monkhorst-Pack mesh of 5×5×5 *k*-points was used for Brillouin zone sampling. While for the calculations of Bi/Gr layers on SiO_2_ substrate, the Perdew–Burke–Ernzerh (PBE) type generalized gradient approximation (GGA) was used for the exchange correlation potentials and a Monkhorst-Pack mesh of 3×3×1 *k*-points was applied. All the structures were relaxed until the Feynman–Hellman forces acting on each atom were less than 0.01 eV Å^−1^.

**4.** **Potential difference between** **Gr and Bi in Bi/Gr and Gr/Bi heterojunctions**


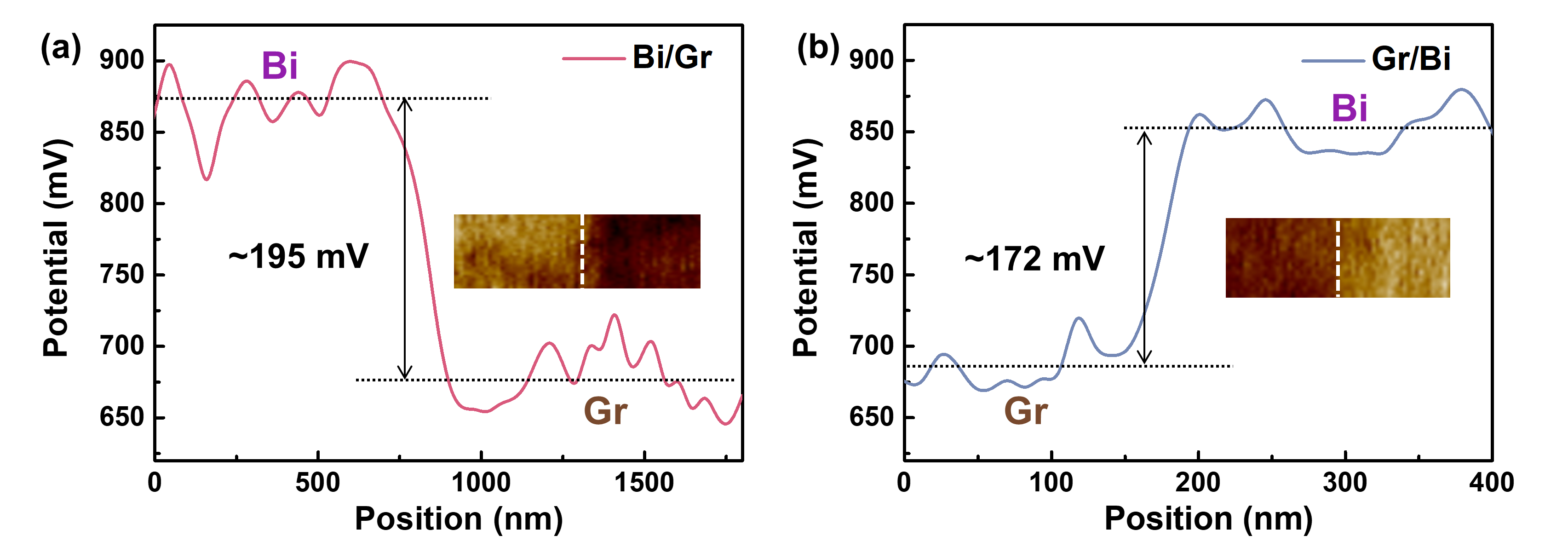


**Figure S4.** Potential difference between Gr and Bi in Bi/Gr and Gr/Bi heterojunctions.

The surface potential difference at Gr and Bi is obtained by Kelvin Probe Force Microscopy (KPFM) measurement, as shown in Figure S4. It is observed that Bi has a higher Fermi energy level than Gr, providing the direct evidence of the existence of a barrier when the heterojunction is formed. Moreover, different stacking order has an influence on the potential difference with the value of 195 mV in Bi/Gr and 172 mV in Gr/Bi due to the substrate effect.

**5.** **Measured THz photoresponse under the light pulse**

To examine the device-to-device variation, we randomly selected 100 devices from each of the Bi/Gr heterostructure, Bi nanofilm, and Gr/Bi heterostructure samples (Figures S5a,c,e). The *MD*(*t*) curves of three systems all exhibit the relatively concentrated distribution, reflecting excellent device uniformity. Furthermore, the corresponding statistical distribution histograms of *MD*(*t*=0) for the 100 devices are well-fitted by Gaussian distributions to confirm the consistency of the device performance (Figures S5b,d,f).


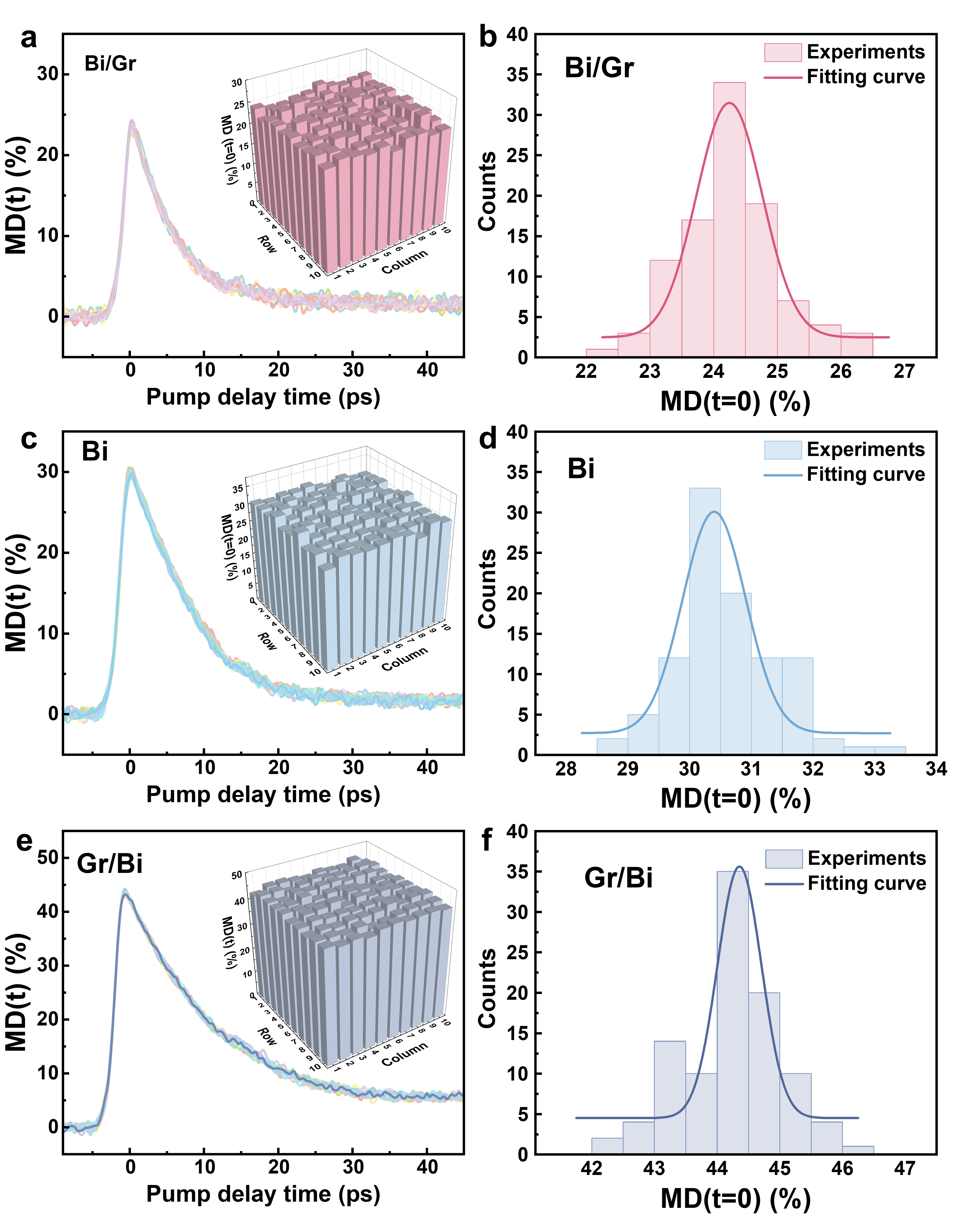


**Figure S5.** (a, c, e) *MD*(*t*) curves of 100 devices randomly selected from Bi/Gr heterostructure, Bi nanofilm, and Gr/Bi heterostructure. Insets: *MD*(*t*=0) histogram of the devices. (b, d, f) Corresponding statistical diagram of *MD*(*t*=0) at pump power of 50 mW. The fitting curve is a Gaussian function.


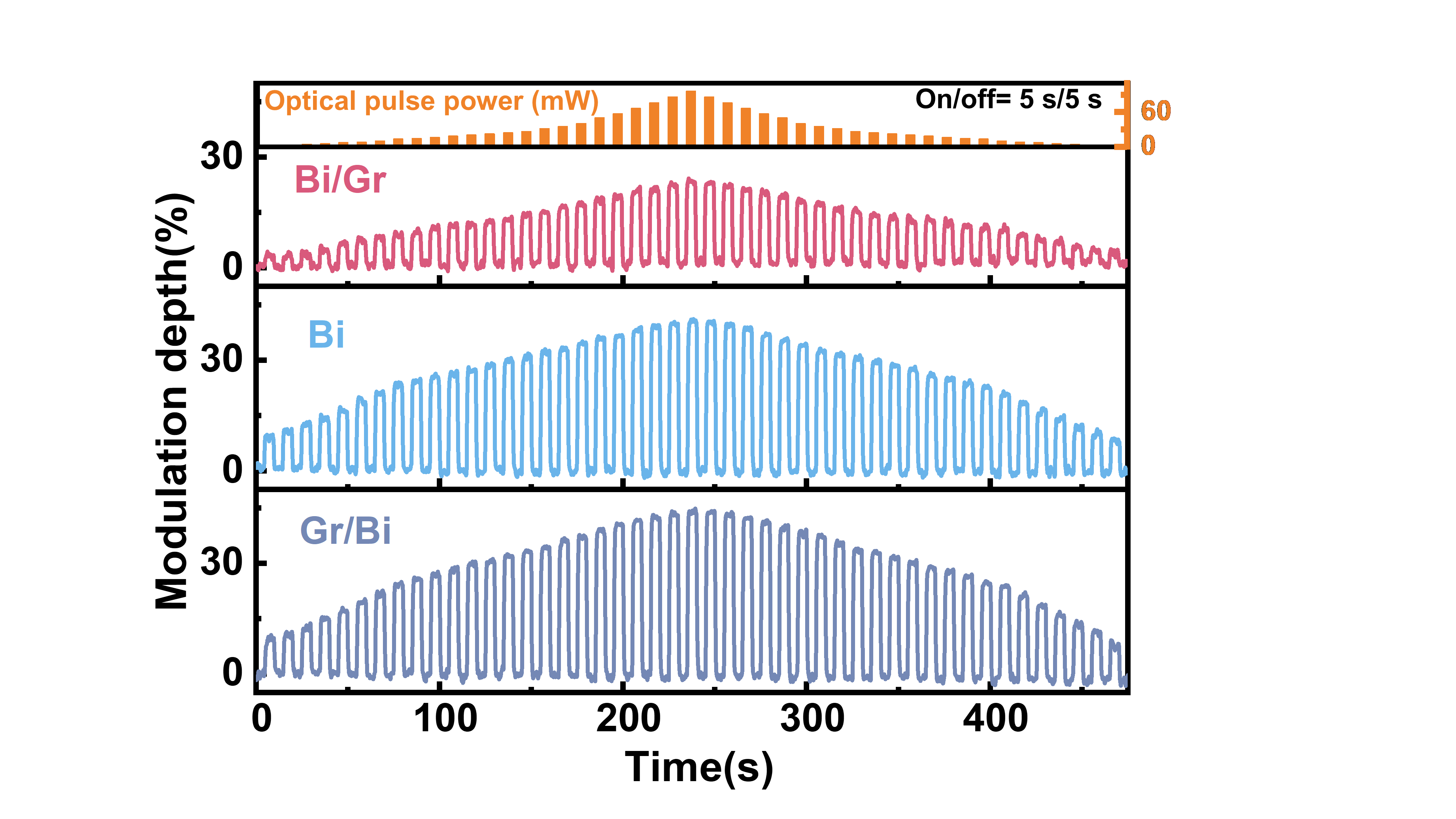


**Figure S6**. Measured THz photoresponse of Bi/Gr heterostructure, Bi nanofilm, and Gr/Bi heterostructure with potentiation and depression synaptic function using incremental programming pulses.


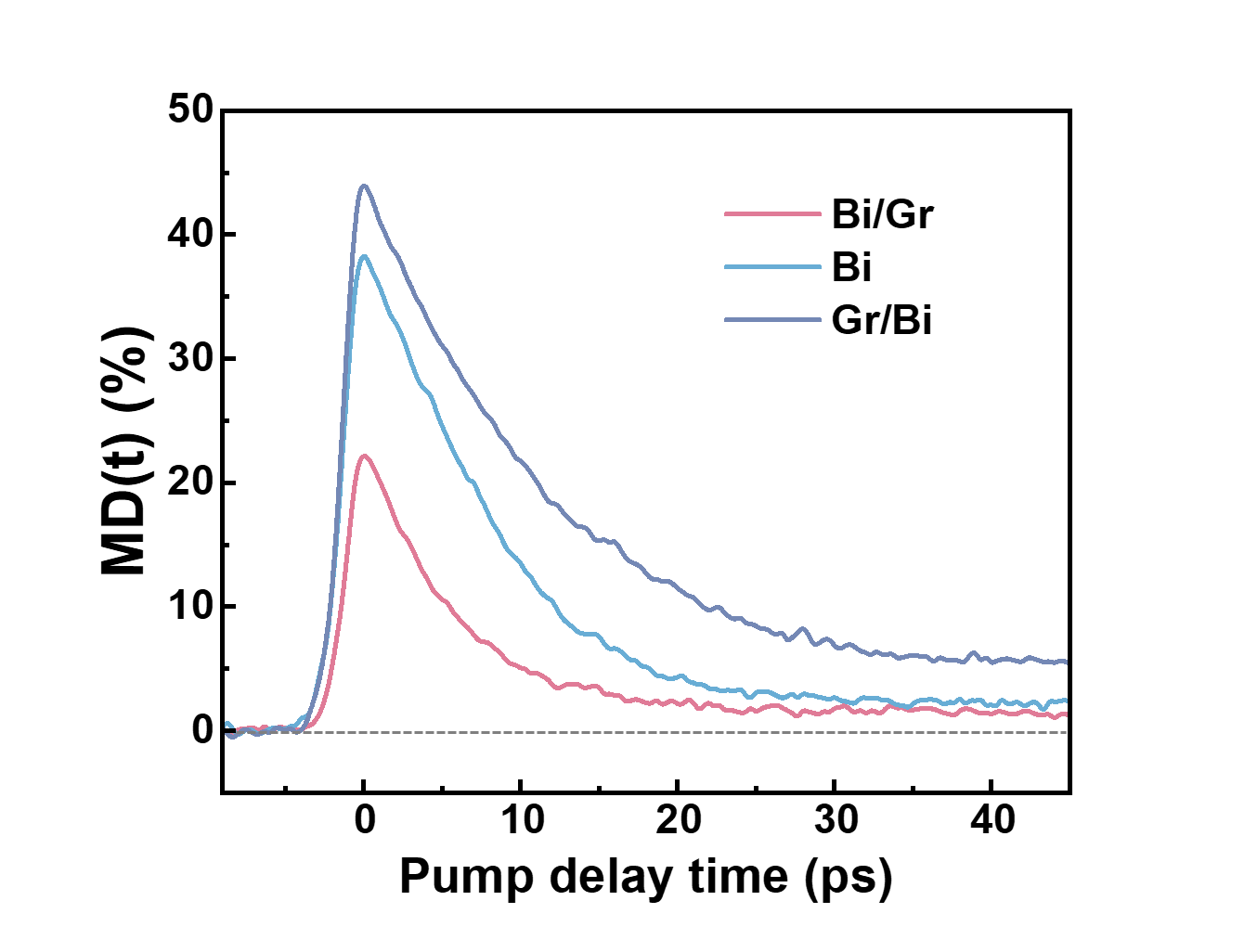


**Figure S7**. Transient *MD*(*t*) curves for Bi/Gr, Bi and Gr/Bi samples after the cessation of 24th optical pulses.

Figure S7 presents the transient evolution of the *MD* for the Bi/Gr heterostructure, Bi nanofilm, and Gr/Bi heterostructure. At pump delay time of 0 ps, the *MD* exhibits a sharp rise and reaches its peak value, attributed to the generation of photocarriers induced by the pump pulse. The pump delay time after 0 ps corresponds to the cessation of the optical pulses, showing that the *MD*(*t*) gradually recovers to the initial state within tens of picoseconds.

**6.** **Details in THz-ONN training**

To define the update rule of weight *w* and bias *b* in our THz-ONN simulations, non-linearity (NL) of experimentally obtained potentiation and depression curves is calculated. The NL values for the potentiation and depression are calculated by fitting potentiation and depression profiles with the followings, respectively^4–7^.

$$\left\{\begin{matrix} G_{\text{n+1}}\text{=}G_{n}+\Delta G_{P}=G_{n}+\alpha_{P}e^{-\beta_{P}\frac{G_{n}-G_{\min}}{G_{\max}-G_{\min}}}\left(\Delta G^{+}>0,G^{+}\uparrow\right) \\ G_{\text{n+1}}\text{=}G_{n}+\Delta G_{D}=G_{n}-\alpha_{D}e^{-\beta_{D}\frac{G_{\max}-G_{n}}{G_{\max}-G_{\min}}}\left(\Delta G^{+}<0,G^{+}\downarrow\right) \end{matrix}\right.$$

Where *G_n_* and *G_n+1_* are the conductance of *n^th^* and (*n*+1)*^th^* pulses, *G_min_* and *G_max_* are the minimum and maximum conductance, *α_P_* and *α_D_* are the differences in the conductance between two points on the potentiation and depression curves, *β_P_* and *β_D_* are the curvatures of the potentiation and depression curves (i.e., NL), respectively. In order to facilitate the fitting of the unknown parameters (*α_P_*, *α_D_*, *β_P_,* and *β_D_*) and the application of conductivity values in THz-ONN, the above equations have been transformed to new forms by transformation and normalization.

$$\left\{\begin{matrix} \ln\left[(G_{\text{n+1}}-G_{n})/G_{\max}\right]=\beta_{P}\frac{G_{\min}-G_{n}}{G_{\max}-G_{\min}}+\ln\alpha_{P} \\ \ln\left[(G_{n}-G_{\text{n+1}})/G_{\max}\right]=\beta_{D}\frac{G_{n}-G_{\max}}{G_{\max}-G_{\min}}+\ln\alpha_{D} \end{matrix}\right.$$

In each backpropagation process, we first calculate the expected change of each *w* and *b* (i.e. Δ*w* and Δ*b*) based on the Batch Gradient Descent. To prevent overfitting, if |Δ*w*|, |Δ*b*|＜5×10^−4^, the conductance of this *w* or *b* will not be updated. Otherwise, the conductance will be updated as follows. Considering that at most one pulse is applied for each device update, there are three ways to update the device conductance, namely, applying an optical pulse, applying an electrical pulse and no pulse.

**7. Comparison of reported device properties based on 2D materials**

As shown in Table S2, most existing devices in the ultraviolet, visible, and near-infrared bands typically operate on second or millisecond timescales. Our work utilizes the unique ultrafast photocarrier dynamics of 2D materials in the THz band to achieve picosecond-scale transient responses, emulating the visual processing of the retina.

**Table S2. Comparison of reported device properties based on 2D materials.**

| **Response time** | **Working band** | **Device materials** | **Modulation mechanism** | **Application** | **Refs** |
| --- | --- | --- | --- | --- | --- |
| Second (s) | Ultraviolet | MoTe_2_ | Charger tunnel/optoelectronics | Logic gate XOR | 8 |
|  |  | MoS_2_ | Charge trap/optoelectronics | Visual adaptation | 9 |
|  | Visible | Graphene/MoSe_2_ | Charge transfer/optoelectronics | Dynamic gesture recognition | 10 |
|  |  | MoS_2_/h-BN | Charger tunnel/optoelectronics | Multi-bit storage ability | 11 |
|  |  | In_2_Se_3_ | Polarization switch/optoelectronics | Mixed color  Pattern Recognition | 12 |
|  |  | WSe_2_ | Polarization switch/ optoelectronics | Information encryption | 13 |
|  |  | In_2_O_3_ | Bidirectional photovoltage-drive/optoelectronics | Motion-color detection | 14 |
|  | Infrared | MoS_2_ | Charge trap/optoelectronics | Motion perception | 15 |
|  |  | PdSe_2_ | Defect trap/optoelectronics | Dynamic trajectory  Perception | 16 |
|  |  | ReSe_2_ | Defect trap/optoelectronics | Polarization-sensitive vision | 17 |
| Millisecond (ms) | Ultraviolet | C_3_N_4_ | Floating gate/optoelectronics | UV-selective retina | 18 |
|  |  | BP | Defect trap/optoelectronics | Digital logic operations | 19 |
|  | Ultraviolet- infrared | InAs | Defect trap/optoelectronics | Optical detection | 20 |
|  | Visible | ReS_2_/h-BN/MoS_2_ | Floating Gate/optoelectronics | Nonvolatile memory | 21 |
|  |  | BP/WSe_2_/h-BN | Charger tunnel/optoelectronics | Motion detection | 22 |
|  |  | graphene/MoS_2_ | Charge transfer/photonics | Image recognition | 23 |
|  |  | Nb_2_GeTe_4_ | Charge trap/optoelectronics | Perception and decision-making | 24 |
|  |  | WS_2_ | Photoluminescent dynamics/ photonics | Visual short-term memory | 25 |
|  | Infrared | Graphene | Charge trapping/optoelectronics | Image noise filtering | 26 |
|  |  | In_2_O_3_/Al_2_O_3_ | Photoelectron trap/optoelectronics | Image recognition | 27 |
| Microsecond (μs) | Visible | ReS_2_/WSe_2_ | Charge trapping/optoelectronics | Multi-bit storage ability | 28 |
| Picosecond (ps) | Terahertz | Bi-based structures | Photocarrier relaxation/photonics | Image recognition | This work |

**References**

1. A. C. Ferrari, and D. M. Basko, Raman Spectroscopy as a Versatile Tool for Studying the Properties of Graphene. *Nat. Nanotechnol.* **2013**, *8*, 235–246.
2. A. Das, S. Pisana, B. Chakraborty, et al., Monitoring Dopants by Raman Scattering in an Electrochemically Top-Gated Graphene Transistor. *Nat. Nanotechnol.* **2008**, *3*, 210–215.
3. X. Xing, Z. Zhang, C. Quan, et al., Tunable Ultrafast Electron Transfer in WSe_2_-Graphene Heterostructures Enabled by Atomic Stacking Order. *Nanoscale* **2022**, *14*, 7418–7425.
4. T. Ahmed, M. Tahir, M. X. Low, et al., Neuromorphic Imaging: Fully Light‐Controlled Memory and Neuromorphic Computation in Layered Black Phosphorus. *Adv. Mater.* **2021**, *33*, 2170074.
5. Y P. Yao, H. Wu, B. Gao, et al., Fully Hardware-Implemented Memristor Convolutional Neural Network. *Nature* **2020**, *577*, 641–646.
6. Y. Zhai, Y. Zhou, X. Yang, et al., Near Infrared Neuromorphic Computing via Upconversion-Mediated Optogenetics. *Nano Energy* **2020**, *67*, 104262.
7. S. Seo, S.-H. Jo, S. Kim, et al., Artificial Optic-Neural Synapse for Colored and Color-Mixed Pattern Recognition. *Nat. Commun.* **2018**, *9*, 5106.
8. K. Li, T. He, N. Guo, T. Xu, X. Fu, F. Wang, H. Xu, G. Li, S. Liu, K. Deng, Y. Xiao, J. Miao, W. Hu, *Adv*. *Opt*. *Mater*. **2023**, *11*, 2202379.
9. F. Liao, Z. Zhou, B. J. Kim, J. Chen, J. Wang, T. Wan, Y. Zhou, A. T. Hoang, C. Wang, J. Kang, J.-H. Ahn, Y. Chai, *Nat*. *Electron*. **2022**, *5*, 84.
10. J. Wang, Y. Lin, J. You, T. Yu, W. Meng, L. Sun, *Adv*. *Intell*. *Syst*. **2025**, *7*, 2401057.
11. H. Lai, Y. Zhou, H. Zhou, N. Zhang, X. Ding, P. Liu, X. Wang, W. Xie, *Adv*. *Mater*. **2022**, *34*, 2110278.
12. Y. Chen, M. Zhang, D. Li, Y. Tang, H. Ren, J. Li, K. Liang, Y. Wang, L. Wen, W. Li, W. Kong, S. Liu, H. Wang, D. Wang, B. Zhu, *ACS Nano* **2023**, *17*, 12499.
13. A. Cao, S. Li, H. Chen, M. Deng, X. Xu, L. Shang, Y. Li, A. Cui, Z. Hu, *Mater*. *Horiz*. **2023**, *10*, 5099.
14. C. Jin, J. Wang, S. Yang, Y. Ding, J. Chang, W. Liu, Y. Xu, X. Shi, P. Xie, J. C. Ho, C. Wan, Z. Zheng, J. Sun, L. Liao, J. Yang, *Adv*. *Mater*. **2025**, *37*, 2410398.
15. M. Huang, X. Liu, F. Yu, J. Li, J. Huang, W. Ali, L. Yang, B. Song, Z. Li, *Adv*. *Mater*. **2025**, *37*, 2412993.
16. H. Gao, X. Jiang, X. Ma, M. Ye, J. Yang, J. Zhang, Y. Gao, T. Li, H. Wang, J. Mei, X. Fu, X. Liu, T. Sun, Z. Guo, P. Guo, F. Chen, K. Zhang, J. Miao, W. Hu, J. Huang, *Nat*. *Commun*. **2025**, *16*, 5241.
17. Y. Zhu, Y. Tao, Z. Wang, J. Bian, Z. Li, M. Qi, Y. Lin, X. Zhao, H. Xu, Y. Liu, *ACS Nano* **2025**, *19*, 25480.
18. H. Park, H. Kim, D. Lim, H. Zhou, Y. Kim, Y. Lee, S. Park, T. Lee, *Adv*. *Mater*. **2020**, *32*, 1906899.
19. L. Xie, X. Chen, Z. Dong, Q. Yu, X. Zhao, G. Yuan, Z. Zeng, Y. Wang, K. Zhang, *Adv*. *Electron*. *Mater*. **2019**, *5*, 1900458.
20. X. Wang, D. Pan, M. Sun, F. Lyu, J. Zhao, Q. Chen, *ACS Appl*. *Mater*. *Interfaces* **2021**, *13*, 26187.
21. Y. Wang, E. Liu, A. Gao, T. Cao, M. Long, C. Pan, L. Zhang, J. Zeng, C. Wang, W. Hu, S. J. Liang, F. Miao, *ACS Nano* **2018**, *12*, 9513.
22. Z. Zhang, S. Wang, C. Liu, R. Xie, W. Hu, P. Zhou, *Nat*. *Nanotechnol*. **2022**, *17*, 27.
23. J. Yu, X. Yang, G. Gao, Y. Xiong, Y. Wang, J. Han, Y. Chen, H. Zhang, Q. Sun, Z. L. Wang, *Sci*. *Adv*. **2021**, *7*, eabd9117.
24. T. Zeng, Z. Zhao, K. Ye, Z. Yu, J. Yan, Y. Zeng, W. Lv, L. Guo, C. Zhao, A. Nie, Z. Zeng, Z. Liu, *Adv*. *Mater*. **2026**, *38*, e09686.
25. F. Ferrarese Lupi, G. Milano, A. Angelini, M. Rosero‐Realpe, B. Torre, E. Kozma, C. Martella, C. Grazianetti, *Adv*. *Funct*. *Mater*. **2024**, *34*, 2470178.
26. C. Han, J. Han, M. He, X. Han, Z. Wu, H. Yu, J. Gou, J. Wang, *Laser Photonics Rev*. **2024**, *18*, 2300976.
27. D. Li, H. Ren, Y. Chen, Y. Tang, K. Liang, Y. Wang, F. Li, G. Liu, L. Meng, B. Zhu, *Adv*. *Funct*. *Mater*. **2023**, *33*, 2303198.
28. K. Liu, T. Zhu, Y. Tang, Y. Zhang, Y. Gu, Y. Zhang, J. Xing, Y. Dong, B. Feng, X. Li, L. Zhang, M. Jiang, H. Xu, *Adv*. *Funct*. *Mater*. **2025**, *35*, e09982.
